# Characterization of a likelihood based method and effects of markers informativeness in evaluation of admixture and population group assignment

**DOI:** 10.1186/1471-2156-6-50

**Published:** 2005-10-14

**Authors:** Bao-Zhu Yang, Hongyu Zhao, Henry R Kranzler, Joel Gelernter

**Affiliations:** 1 Yale University School of Medicine, Department of Psychiatry, New Haven, CT, USA; 2 VA CT Healthcare Center, West Haven, CT, USA; 3 Yale University School of Medicine, Departments of Epidemiology and Public Health, and Genetics, New Haven, CT, USA; 4 University of Connecticut Health Center, Farmington, CT, USA

## Abstract

**Background:**

Detection and evaluation of population stratification are crucial issues in the conduct of genetic association studies. Statistical approaches useful for understanding these issues have been proposed; these methods rely on information gained from genotyping sets of markers that reflect population ancestry. Before using these methods, a set of markers informative for differentiating population genetic substructure (PGS) is necessary. We have previously evaluated the performance of a Bayesian clustering method implemented in the software STRUCTURE in detecting PGS with a particular informative marker set. In this study, we implemented a likelihood based method (LBM) in evaluating the informativeness of the same selected marker panel, with respect to assessing potential for stratification in samples of European Americans (EAs) and African Americans (AAs), that are known to be admixed. LBM calculates the probability of a set of genotypes based on observations in a reference population with known specific allele frequencies for each marker, assuming Hardy Weinberg equilibrium (HWE) for each marker and linkage equilibrium among markers.

**Results:**

In EAs, the assignment accuracy by LBM exceeded 99% using the most efficient marker FY, and reached perfect assignment accuracy using the 10 most efficient markers excluding FY. In AAs, the assignment accuracy reached 96.4% using FY, and >95% when using at least the 9 most efficient markers. The comparison of the observed and reference allele frequencies (which were derived from previous publications and public databases) shows that allele frequencies observed in EAs matched the reference group more accurately than allele frequencies observed in AAs. As a result, the LBM performed better in EAs than AAs, as might be expected given the dependence of LBMs on prior knowledge of allele frequencies. Performance was not dependent on sample size.

**Conclusion:**

The performance of the LBM depends on the efficiency and number of markers, and depends greatly on how representative the available reference allele frequencies are for those of the population being assigned. This method is of value when the parental population is known and relevant allele frequencies are available.

## Background

Population stratification is a crucial issue in conducting genetic association studies, in particular, for case-control study designs, such that if it is not accounted for study results could be invalid – either false positive or false negative [1]. Methods to address the issue have been proposed [2-17]. Before using these methods, an informative set of markers is necessary; this is known as a set of ancestry informative markers (AIMs). In this study, we implemented a likelihood based method (LBM), as an alternative to popular Bayesian methods such as that implemented in STRUCTURE [3,13], and used it to evaluate the informativeness of a selected marker panel and to assess potential for stratification in a sample of European Americans (EAs) and African Americans (AAs) that are known to be admixed.

Likelihood-based methods (LBMs) provide a framework for assignment of individuals to specific populations based on observed allele frequencies in AIMs. LBMs for the classification of individuals into subgroups can be implemented by calculating the probability of a marker genotype profile (i.e., a set of genotypes) based on observations in a reference population with known specific allele frequencies for each marker ("training frequencies"), assuming Hardy Weinberg equilibrium (HWE) for each marker and linkage equilibrium among markers [18]. The LBM method is also called an "assignment test" and is widely applied in molecular ecology and animal forensics for identifying population genetic substructures for animals or plants [18-24]. Research on the assignment test or LBM has not yet focused on the performance of the test or of specific markers in differentiating the PGS in human subjects. In theory, LBM may be better for probabilistic classification of individuals to subpopulations, if certain conditions are met. The most important of these conditions is availability of an accurate set of training frequencies. Obviously, this method may be applicable only if the populations from which the sample to be classified are already known or can be determined. This condition can be met in most situations; for example, the AA population is well known to have principally African and European American ancestry.

In the present study, we compared the performance of LBMs to that of the popular Bayesian approach used by the software program STRUCTURE. We predicted that, if the conditions for successful LBM application are met, LBMs would be more efficient that Bayesian methods for population group assignment, because they make use of more information (i.e., known ancestral population allele frequencies, which are provided *a priori *rather than inferred from the data presented to the program).

## Results

**Figure 1:**
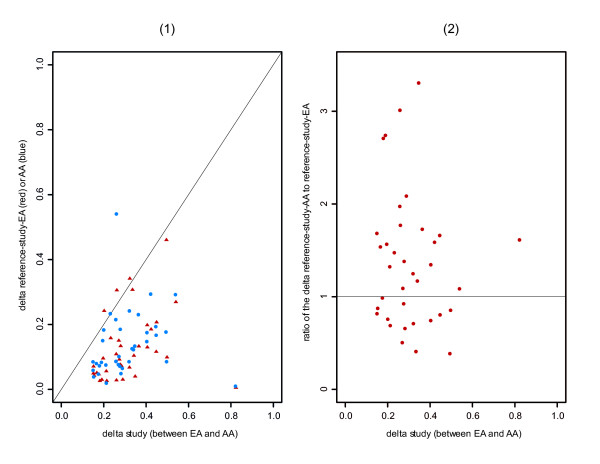
**Marker efficiency in terms of the metric *δ*. **(1) Comparison of delta between for AAs and EAs as observed in our sample, and as reported in the prior literature: *δ*_*study*-*AA*-*EA *_versus *δ*_*reference*-*study*-*EA *_(red triangle) or *δ*_*reference*-*study*-*AA *_(blue dot). (2) Ratio for deltas for each marker (AA/EA) as observed in our sample compared to the prior literature: *δ*_*study*-*AA*-*EA *_versus the ratio of *δ*_*reference*-*study*-*AA *_to *δ*_*reference*-*study*-*EA*_.

We calculated the measure of marker efficiency by the metric *δ *for each marker. (Note that *δ *as defined here is different from that defined in Rosenberg et al. 2003 [25]). We designated *δ*_*study*-*AA*-*EA *_as the measure of marker efficiency between EA and AA in our study populations, and *δ*_*reference*-*study*-*EA *_or *δ*_*reference*-*study*-*AA *_as the quantitative difference in efficiency between marker characteristics as they were reported previously, and as we observed them in the study populations. We observed that the maximum *δ*_*study*-*AA*-*EA *_was 0.82, for the marker FY, and the minimum *δ*_*study*-*AA*-*EA *_was 0.15. The mean was 0.32 and median was 0.28. Larger observed *δ*_*study*-*AA*-*EA *_corresponded to greater marker efficiency for differentiating the EA and AA study populations. Furthermore, smaller values of the *δ*_*reference*-*study *_(including *δ*_*reference*-*study*-*EA *_or *δ*_*reference*-*study*-*AA*_) indicate that the marker as observed is more similar to the marker as described in the reference (and therefore the reported allele frequencies were relatively accurate for LBM training). For markers with higher values of this measure, since they did not match the training frequencies as well, their utility in practice was reduced. An efficient classification marker would be one with bigger *δ*_*study*-*AA*-*EA *_and smaller *δ*_*reference*-*study *_when the reference allele frequencies are used for training for the LBM. Figure 1 shows the relationship of these three *δ *measures; the straight line in the Figure 1(1) indicates the equality of *δ*_*study*-*AA*-*EA *_and *δ*_*reference*-*study*_. Thus, Figure 1(1) illustrates that the majority of the markers have *δ*_*study*-*AA*-*EA *_> *δ*_*reference*-*study*_, and Figure 1(2) shows the ratio of *δ*_*reference*-*study*-*AA *_to *δ*_*reference*-*study*-*EA *_with a horizontal line specifying *δ*_*reference*-*study*-*AA *_= *δ*_*reference*-*study*-*EA*_. (Twenty-two of 36 markers studied (61%) are above the horizontal line, which indicates that they are less representative (of prior reports) for AAs than for EAs. This reduced correspondence of the observed AA allele frequency compared to the prior reports relative to our observations in EA populations, also causes decreased assignment accuracy in AAs compared to EA – in fact, the assignment accuracy in AAs never reaches 100%. Even with imperfect training frequencies, the LBM using the selected makers to classify individuals into subpopulations still performed very well, with average assignment accuracy of 96.8% and 99.9% for AA and EA respectively.) These results illustrate, further, that the selected marker panel is a relatively informative marker set in differentiating between EAs and AAs.

### Assignment accuracy

**Figure 2:**
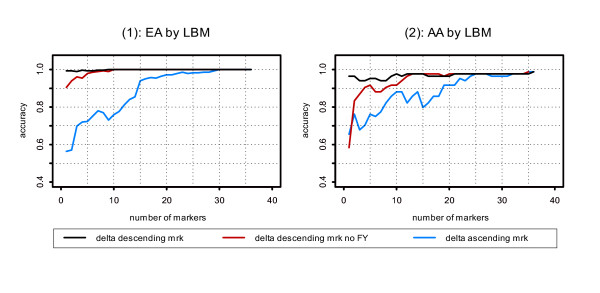
**Assignment accuracy by LBM. **Assignment accuracy by LBM. The markers are adding one by one either by *δ *descending or ascending. Assignment accuracy without FY, the most efficient marker in the panel studied, was also evaluated.

**Figure 3:**
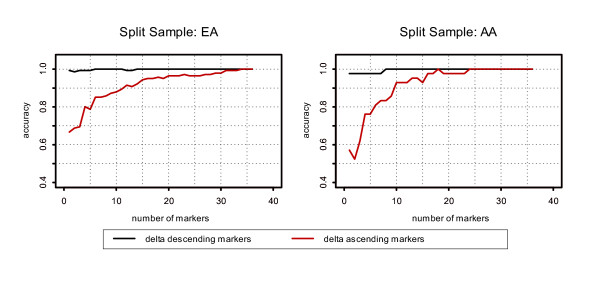
**Assignment accuracy by LBM for Split samples. **Assignment accuracy by LBM for Split samples. Split samples were used to evaluate the impact of reference group of allele frequencies on the assignment accuracy by LBM.

In order to ascertain the smallest sufficient marker set and identify how many makers are needed to reach reasonable assignment accuracy, we took the approach of selecting markers by marker efficiency, as we did previously in evaluating the Bayesian method [1]. The relative assignment accuracy was evaluated by adding markers one-by-one up to 36 markers, with the order of *δ *either descending or ascending; the results are shown in Figure 2 (This result by LBM can be compared with results from STRUCTURE in Yang et al. 2005 [1]; cf. Figure 3, p. 308). FY was the most informative marker, and due to its unique value in distinguishing the EA and AA populations under study, we performed analyses separately either including or excluding this marker.

In EAs (Figure 2, (1)), the assignment accuracy by LBM exceeded 99% using the most efficient marker FY, and reached 100% using the 10 most efficient markers excluding FY (when FY was excluded, the assignment accuracy using the next most efficient marker D11S936 dropped by 9%). In contrast, it would take 29 markers to reach >99% assignment accuracy when the least efficient markers are selected or the seven most efficient markers are omitted. In AAs (Figure 2, (2)), the assignment accuracy reached 96.4% using FY, and then the assignment accuracy changed inconsistently as more markers were added up to 21 markers, at which point assignment accuracy stabilized at 97.6%, achieving the maximum of 98.8% when all 36 markers were used. Overall, using LBM, it can exceed 95% when using at least the 9 most efficient markers. When FY was excluded, the assignment accuracy dropped by 38%.

This 38% drop, which reflects the difference in accuracy between the most efficient marker, FY, and the second most efficient one, D11S936, was further investigated by a corresponding analysis in which the study sample was randomly split into two groups and one group was treated as a reference sample. The drop declined to 6%, which was more comparable to the 9% in EAs. Thus, this reduced accuracy was in large part attributable to mismatch between reported training allele frequencies and frequencies that are more representative of our Northeastern US AA population. LBM never reaches perfect assignment accuracy for AAs in this sample even when all the 36 markers were used, but accuracy did reach 98.8%.

### Comparison of observed and reference allele frequencies

The high assignment accuracy by LBMs was observed notwithstanding the deviation between our observed allele frequencies and the reference frequencies described above. We further compared our observed allele frequencies with published reference allele frequencies using the *χ*^2 ^test. In EAs, after adjusting for sample size, there were 19 markers that differed at p < 0.05, while in AAs, the corresponding number of markers was 29. In other words, allele frequencies observed in EAs matched the reference group more closely than did allele frequencies observed in AAs. As a result, the LBM performed better in EAs than AAs, as might be expected given the dependence of LBMs on prior knowledge of allele frequencies.

### Evaluation of the influence of mismatched reference allele frequencies on assignment accuracy by means of split samples

As noted above, in many cases our observed allele frequencies showed nominally significant differences from population reference frequencies. This could reflect, for example, sampling error, or differences in allele frequency for population groups with similar self-identified ethnicity that are assessed at different geographic locations. To further assess the impact of the reference group on the assignment accuracy for LBM, we randomly split our EA and AA study datasets each into two equal-sized samples, treating one as the study group and the other as the reference group. Thus, we were able to model geographically appropriate allele frequencies for each group, at the expense of reducing the analysis sample size by a factor of two. The distributions of the allele frequencies for the two split samples are the same in EAs and AAs for all the markers based on the *χ*^2 ^test (p-value ranges from > 0.57 to 1). The results (Figure 3) for AAs using internal split samples improved dramatically compared to the results using the external reference group in AAs (Figure 2). These results (Figure 3) illustrate that the performance of the LBM depends greatly on how representative the reference allele frequencies are to those of the population being assigned when the parental population is known.

### Logarithm likelihood ratio

**Figure 4:**
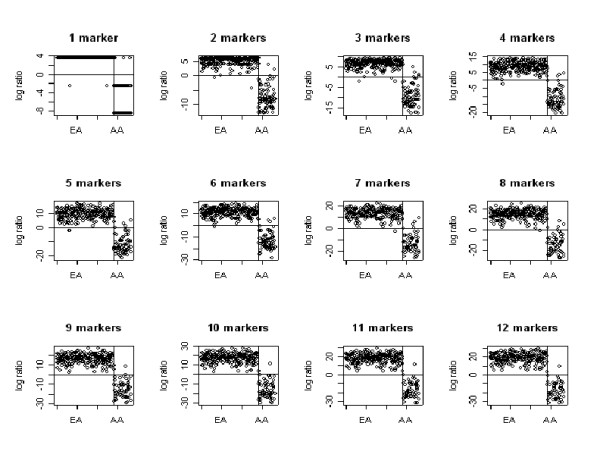
**Logarithm of likelihood ratio for each individual. **Logarithm of likelihood ratio for each individual grouping by their self-identified ethnicity. Markers were added one by one with *δ *descending. The first marker is FY.

We also calculated the logarithm of the likelihood ratio, expressing the comparison of the probability of being in the EA group compared to the AA group, based on formula (2) (Methods section), and generated a visual display of correct or misplaced group assignment for each individual, adding the markers one by one using a descending value of *δ*. Figure 4 shows the 12 most efficient markers. The horizontal line represents a log likelihood ratio of zero; those above zero are allocated to EA, and below zero to AA (refer to equation (2)). The vertical line separates the groups. Therefore, those in the upper right and lower left quadrants are misclassified based on self-identified race. The first graph represents the allocation of each individual using only the most efficient marker, FY. As markers are added to the analyses, the log likelihood ratios increase and the separation between clusters become more and more marked. (Note that the Y-axis scale is not constant.)

One individual in the AA series appeared to be misclassified; see Figure 4 with 9 to 12 markers. Based on this observation, we examined the phenotypic information for this subject, and determined that, although self-identified as AA, the subject had one AA and one EA parent.

### Comparison of LBM results with Bayesian results obtained using STRUCTURE

**Figure 5:**
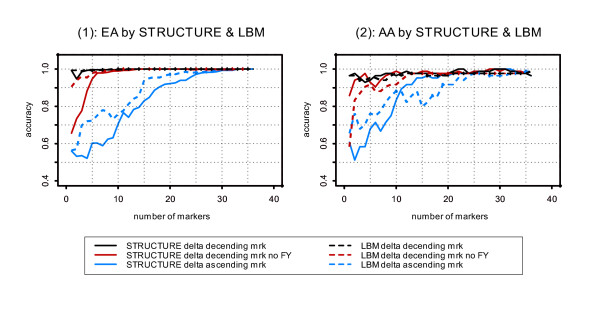
**Comparison of STRUCTURE and LBM on assignment accuracy. **The markers are adding one by one either by delta descending or ascending. Assignment accuracy without FY, the most efficient marker in the panel studied, was also evaluated.

We compared the performance of LBM with results obtained using STRUCTURE and the same panel of markers by Yang et al. 2005 [1] (Figure 5); the samples used for Figure 5 are exactly the same as those for Figure 3 in Yang et al. 2005 [1] (cf. Figure 3, p. 308). In EAs (Figure 5 – (1)), the LBM provided more accurate group assignment than STRUCTURE, with the FY locus included or excluded. In AAs (Figure 5 – (2)), the relative performance of STRUCTURE and LBM was mixed.

## Discussion

The LBM is appealing for population group assignment because it is straightforward and easily implemented, provided that sufficiently accurate reference allele frequencies are available. We provide a set of allele frequencies for all markers herein that will prove useful for classifying populations similar to those discussed in the present article [see Additional file 1]. Under these circumstances, the LBM should classify individuals at least as accurately as STRUCTURE, and probably more accurately. However, a representative reference population may be difficult to establish in some cases. With a good reference group, as shown in the analysis of split samples (p-values of the *χ*^2 ^test range from >0.57 to 1 for distributions of allele frequencies for the two split samples), LBM performed very well. In EAs, the clustering by LBM is as good as by STRUCTURE (using an ancestry model of admixture and an allele frequency dependence model) for *δ *descending, but LBM performs better for ascending values of *δ*. For AAs, LBM and STRUCTURE cluster the groups equally well. STRUCTURE retains certain advantages, such as the ability to classify individuals by proportional ancestry for subsequent application of the structured association method, as discussed elsewhere [1]. It should be noted that the superior performance of LBMs over STRUCTURE, when observed, depends on LBM having more data available than STRUCTURE in the form of reference allele frequencies.

The observed allele frequencies in this study matched reference allele frequencies better for EA than AA populations. Subjects from some populations from different geographic areas might have quite different admixture proportions and ancestral origins. This is demonstrably the case for African-Americans, since in different parts of the US the percent admixture from EAs is known to range at least from 12% to 23% [26]. Another issue with LBM involves justification for the multiplication of allele frequencies across loci under the assumption of linkage equilibrium. If the allele frequencies of different STRs vary among subpopulations, then the loci are not in complete linkage equilibrium or are not statistically independent even if they are genetically unlinked. However, we did assume linkage equilibrium within the subpopulations. This is also an underlying assumption for STRUCTURE [4]. This assumption might prove to be problematic under some circumstances, but the practical impact seemed minimal for the present study, as evidenced by the fact that LBM performed well.

The result from the single most informative marker, FY, could exceed 99% and 96% assignment accuracy in EAs and AAs, respectively. This result is, of course, sample-specific to some extent; AA subjects who are homozygous for the allele more characteristic of European ancestry (i.e., FY (+/+)), should have a population frequency of about 4%, given a 20% admixture rate from EA, and would be misclassified into the EA group if based only on this marker; this misclassification rate is equal to what we observed, about 4% in AAs. Likewise, EAs heterozygous for the FY(-) allele characteristic of AAs are observed as well, and they are liable to be misclassified as AAs. Our Northeastern US AA population had approximated the expected European admixture rate, based on the information from FY.

The sample size of the populations being assigned is not an issue for LBM, while it is for STRUCTURE. The Bayesian cluster approach taken by STRUCTURE requires building a likelihood function from the observed samples to infer allele frequencies, such that if the sample size is insufficient, the estimated allele frequencies might not be accurate. As a result, sample size in each subgroup affects the assignment accuracy, and our simulation result [1] shows that approximately fifty subjects are required to have stable assignment accuracy by STRUCTURE. LBM, in contrast, uses allele frequencies from the reference populations; there is no need to estimate allele frequencies by LBM. Thus, even a single individual can be assigned accurately using the LBM.

We conclude that assignment accuracy by LBM depends on the efficiency of the markers selected (FY alone can separate EAs and AAs with accuracy that can approach 99% for excluding AAs from a presumed EA sample), the number of markers (other things being equal, more markers produce higher assignment accuracy), and greatly on how representative the parental population reference allele frequencies are for the populations being queried.

## Methods

### Subjects

Three hundred sixty-six individuals recruited in the Northeastern US (classified as 282 EAs, 84 AAs) were studied. These individuals were selected from a larger sample for evaluation of this likelihood based method because they had complete marker data for all markers described below. All subjects provided informed consent as approved by the appropriate institutional review boards.

### Markers genotyped

Detailed marker and genotyping information was described previously [1]. Briefly, two different sets of STR markers were used. First, we used the AmpFLSTR Identifiler PCR Amplification Kit (Applied Biosystems (ABI), Foster City, CA), which provides data from a set of 16 loci useful for forensic purposes (D8S1179, D21S11, D7S820, CSF1PO, D3S1358, TH01, D13S317, D16S539, D2S1338, D19S443, vWA, TPOX, D18S51, D5S818, FGA, and amelogenin). Amelogenin is used for sex identification rather than for polymorphism content, so information from that locus was not included in any analyses. Second, we selected 21 markers known to have high *δ *between EAs and AAs, and in some cases Hispanic and Asian populations, based on the report of Smith et al. 2001 [27]. This marker panel includes markers D1S196, D1S2628, D2S162, D2S319, D5S407, D5S410, D6S1610, D7S640, D7S657, D8S272, D8S1827, D9S175, D10S197, D10S1786, D11S935, D12S352, D14S68, D15S1002, D16S3017, D17S799, and D22S274. We also genotyped marker FY, added to the 36 STRs because of its known value in identifying individuals of primarily African ancestry.

### Measures of marker efficiency

*δ *was used to measure the marker efficiency. The definition and properties of *δ *are described in Yang et al. 2005 [1]. Briefly, *δ *is half the sum of the absolute difference in population frequency over all alleles for each marker between two populations.

### Analysis with the likelihood-based method (LBM)

We assumed HWE among alleles for each marker within populations and linkage equilibrium between markers. The likelihood, or the probability of observed genotype profile, for each individual to be in a specific population is calculated as



where *X *is a vector of genotypes of marker loci, *Z *is the proposed population of origin, *P*_*Z*_(*p*_11_, *p*_12_,..., ) is the set of reference allele frequencies *p*_11_, *p*_12_,...,  for the *n*_*m *_alleles of m markers of population *Z*, and *h *is a dummy variable for homozygosity (i.e., when the locus is homozygous, *h *is 1, otherwise *h *is 0) for each marker locus. When an allele is absent for a given population in the reference frequencies, the corresponding allele frequency in the study group is estimated and used in the calculation of likelihood. An individual is assigned to a population if the maximum likelihood results from assignment to that population among all possible population-specific likelihoods calculated. For assigning individuals into one of two populations *A *or *B*, an individual is assigned to population *A *if the logarithm of likelihood ratio is greater than zero, or otherwise to *B*, as shown in equation (2).



An individual was considered to be assigned accurately to a group when the greatest likelihood among all the calculated likelihoods assigned an individual the same ethnicity as the self-identified population group of that individual. Assignment accuracy in each population group was defined as the proportion of correctly assigned ethnicities. (The above decision rule is optimal if we have equal priors of proportion for the two ethnic groups. However, when there are more people from one group, *a priori*, then the prior information needs to be incorporated to improve the overall performance in terms of misclassification rate.) The method was realized in R/S-Plus; the function codes are available upon request from the authors.

The initial set of reference population-specific allele frequencies (training frequencies) for the 36 markers were derived from ABI reference materials [27] or Smith et al. 2001 [28], depending on the source of each marker. Since ABI uses different nomenclature (in some cases) and we redesigned some primers referenced by Smith to facilitate efficient genotyping, each observed allele had to be matched to the corresponding allele for each marker. Alleles at one marker locus (D6S1610) described by Smith et al. 2001 [28] could not be matched accurately to our data from the same marker (however, the value of EA/AA*δ *that we derived for that marker, 0.336, was similar to the value reported by Smith et al., which was 0.337). The *χ*^2 ^test was used to compare the allele distributions of the study group and the reference group.

### Evaluation of the impact of the training frequencies on population group assignment accuracy

To evaluate the impact of the training frequencies on population group assignment accuracy, we compared the literature-derived training allele frequencies (described above) with training allele frequencies computed from our specific populations. To do so, we randomly split the 282 EAs and 84 AAs into two equal-sized groups. One was treated as the study group, and the other was treated as the reference group, from which the training allele frequencies were estimated.

## Authors' contributions

BZY participated in study design, wrote the computer code, carried out the statistical analyses and drafted the manuscript. HZ participated in study design, and conceptual and technical assistance. HRK provided sample recruitment and phenotyping, and commented on the manuscript. JG designed the study, coordinated genotyping efforts and helped draft the manuscript. All authors read and approved the final manuscript.

## Supplementary Material

Additional File 1 Population allele frequencies in the EA and AA samples for the 35 STRs markers and the FY marker studied. For each marker, the marker name and alleles are listed with the allele frequencies. Click here for file
