# Supplementary material for: Characterization of a likelihood based method and effects of markers informativeness in evaluation of admixture and population group assignment
Source: BMC Genet. 2005 Oct 14;6:50. doi: 10.1186/1471-2156-6-50 (PMC1285360; doi:10.1186/1471-2156-6-50)
Supplement: Additional File 1 — Population allele frequencies in the EA and AA samples for the 35 STRs markers and the FY marker studied. For each marker, the marker name and alleles are listed with the allele frequencies. [file 1471-2156-6-50-S1.doc]

## Additional file 1

[1]:CSF1PO

Allele 309 313 317 321 325 329 333 337

EA 0.002 0.004 0.012 0.287 0.287 0.330 0.071 0.007

AA 0.065 0.065 0.042 0.226 0.274 0.292 0.030 0.006

[2]:D2S1338

Allele 304 312 316 320 324 328 332 336 340 344 EA 0.000 0.057 0.223 0.071 0.122 0.144 0.034 0.027 0.094 0.115

AA 0.042 0.137 0.036 0.185 0.077 0.149 0.125 0.083 0.089 0.065

Allele 348 352 356

EA 0.101 0.009 0.004

AA 0.006 0.006 0.000

[3]:D3S1358

Allele 108 116 120 124 128 132 136 140

EA 0.002 0.004 0.151 0.222 0.277 0.206 0.137 0.004

AA 0.000 0.012 0.101 0.327 0.304 0.220 0.036 0.000

[4]:D5S818

Allele 139 143 147 151 155 159 163 167

EA 0.009 0.039 0.053 0.333 0.367 0.184 0.011 0.004

AA 0.054 0.024 0.030 0.220 0.351 0.298 0.012 0.012

[5]:D7S820

Allele 255 259 263 267 268 271 275 279 283 287

EA 0.000 0.014 0.160 0.165 0.002 0.261 0.213 0.138 0.046 0.002

AA 0.006 0.012 0.238 0.125 0.000 0.357 0.167 0.083 0.012 0.000

[6]:D8S1179

Allele 124 128 132 136 140 144 148 152 156 160

EA 0.016 0.016 0.094 0.059 0.138 0.362 0.174 0.112 0.025 0.005

AA 0.006 0.012 0.048 0.024 0.155 0.167 0.304 0.214 0.060 0.012

[7]:D13S317

Allele 212 216 220 224 228 232 236 240 244

EA 0.002 0.131 0.074 0.067 0.348 0.264 0.082 0.030 0.002

AA 0.000 0.006 0.030 0.030 0.274 0.470 0.143 0.048 0.000

[8]:D16S539

Allele 264 268 272 276 280 284 288

EA 0.025 0.105 0.064 0.30 0.296 0.186 0.025

AA 0.006 0.196 0.149 0.25 0.220 0.149 0.030

[9]:D18S51

Allele 271 275 277 279 283 287 291 295 299 303 EA 0.002 0.007 0.000 0.016 0.14 0.101 0.179 0.115 0.128 0.128

AA 0.006 0.006 0.012 0.06 0.054 0.054 0.143 0.137 0.179 0.125

Allele 307 311 315 319 327

EA 0.105 0.044 0.025 0.007 0.004

AA 0.000 0.137 0.065 0.024 0.000

[10]:D19S433

Allele 105 109 113 115 117 119 121 123 125 127 EA 0.000 0.002 0.090 0.002 0.243 0.009 0.353 0.014 0.172 0.046

AA 0.071 0.107 0.012 0.286 0.048 0.226 0.060 0.060 0.077 0.012

Allele 129 131 133 135

EA 0.044 0.018 0.005 0.002

AA 0.006 0.036 0.000 0.000

[11]:D21S11

Allele 192 196 200 202 204 206 208 210 212 214 EA 0.000 0.051 0.177 0.002 0.199 0.002 0.254 0.030 0.064 0.101

AA 0.089 0.268 0.000 0.155 0.000 0.167 0.024 0.077 0.042 0.006

Allele 216 218 220 222 224 226 228 232

EA 0.016 0.004 0.000 0.000 0.083 0.000 0.018 0.000

AA 0.006 0.006 0.042 0.012 0.060 0.006 0.024 0.018

[12]:FGA

Allele 210 214 218 220 222 226 228 230 234 236 EA 0.002 0.002 0.021 0.000 0.078 0.122 0.002 0.142 0.188 0.012

AA 0.000 0.000 0.012 0.060 0.054 0.000 0.119 0.161 0.000 0.161

Allele 238 254 258 262 266 240 242 246 250

EA 0.156 0.009 0.004 0.000 0.000 0.005 0.160 0.073 0.025

AA 0.006 0.036 0.060 0.006 0.006 0.000 0.185 0.089 0.048

[13]:TH01

Allele 167 171 175 179 183 186 187 191

EA 0.004 0.225 0.170 0.113 0.167 0.307 0.012 0.002

AA 0.012 0.161 0.387 0.214 0.143 0.083 0.000 0.000

[14]:TPOX

Allele 222 226 230 234 238 242 246

EA 0.004 0.004 0.546 0.092 0.051 0.27 0.034

AA 0.042 0.024 0.381 0.208 0.083 0.22 0.042

[15]:vWA

Allele 154 158 162 166 170 174 178 182 186 190

EA 0.000 0.000 0.000 0.131 0.094 0.184 0.273 0.211 0.089 0.016

AA 0.006 0.006 0.006 0.083 0.256 0.226 0.220 0.107 0.077 0.012

Allele 194

EA 0.002

AA 0.000

[16]:D2S319

Allele 120 126 128 130 132 134 136

EA 0.000 0.121 0.046 0.349 0.257 0.213 0.014

AA 0.006 0.149 0.101 0.220 0.113 0.375 0.036

[17]:D17S799

Allele 185 187 189 191 193 195 197 199 201 203

EA 0.000 0.007 0.346 0.012 0.057 0.232 0.034 0.179 0.023 0.105

AA 0.006 0.149 0.167 0.024 0.226 0.137 0.113 0.125 0.024 0.030

Allele 205

EA 0.005

AA 0.000

[18]:D8S272

Allele 237 239 241 243 245 247 249 251 253 255

EA 0.032 0.002 0.117 0.014 0.401 0.053 0.011 0.174 0.027 0.115

AA 0.042 0.006 0.054 0.018 0.196 0.244 0.054 0.161 0.119 0.071

Allele 257 259 261

EA 0.044 0.005 0.005

AA 0.036 0.000 0.000

[19]:D1S196

Allele 320 322 326 328 330 332 334 336

EA 0.344 0.027 0.229 0.071 0.225 0.101 0.004 0.000

AA 0.333 0.018 0.060 0.095 0.351 0.089 0.048 0.006

[20]:D7S640

Allele 106 108 110 112 114 116 118 120 122 124

EA 0.00 0.000 0.002 0.012 0.032 0.195 0.060 0.179 0.165 0.129

AA 0.06 0.024 0.000 0.149 0.006 0.101 0.048 0.071 0.161 0.065

Allele 126 128 130 132 134 136 138 140 142 144

EA 0.082 0.039 0.030 0.027 0.011 0.014 0.012 0.004 0.002 0.004

AA 0.048 0.054 0.048 0.071 0.071 0.018 0.000 0.000 0.006 0.000

Allele 146

EA 0.002

AA 0.000

[21]:D8S1827

Allele 156 158 160 162 164 166

EA 0.051 0.023 0.530 0.037 0.332 0.027

AA 0.036 0.327 0.256 0.054 0.256 0.071

[22]:D7S657

Allele 246 248 250 252 254 256 258 260 262 264 EA 0.000 0.055 0.055 0.176 0.037 0.005 0.044 0.092 0.440 0.082

AA 0.071 0.101 0.351 0.274 0.030 0.006 0.024 0.042 0.065 0.030

Allele 266 268 272

EA 0.007 0.005 0.002

AA 0.006 0.000 0.000

[23]:D22S274

Allele 272 276 278 280 282 284 286 288 290 292

EA 0.004 0.002 0.007 0.101 0.273 0.289 0.105 0.106 0.103 0.005

AA 0.083 0.000 0.000 0.119 0.095 0.363 0.083 0.113 0.119 0.006

Allele 294

EA 0.005

AA 0.018

[24]:D14S68

Allele 308 310 314 316 318 320 322 324 326 328 EA 0.002 0.000 0.002 0.011 0.129 0.041 0.044 0.305 0.186 0.121

AA 0.000 0.024 0.101 0.024 0.095 0.119 0.071 0.232 0.220 0.060

Allele 330 332 334 336

EA 0.113 0.037 0.007 0.002

AA 0.042 0.012 0.000 0.000

[[25]]:D5S407

Allele 84 86 88 90 92 94 96 98 100 102 EA 0.156 0.018 0.112 0.041 0.028 0.090 0.048 0.152 0.218 0.106

AA 0.042 0.065 0.244 0.030 0.095 0.054 0.065 0.095 0.149 0.060

Allele 104 106 108

EA 0.021 0.004 0.005

AA 0.071 0.024 0.006

[26]:D2S162

Allele 114 116 120 122 124 126 128 130 132 134 EA 0.000 0.004 0.103 0.004 0.000 0.004 0.032 0.053 0.332 0.142

AA 0.012 0.006 0.125 0.012 0.054 0.101 0.107 0.095 0.274 0.107

Allele 136 138 140 142 144 146

EA 0.105 0.050 0.142 0.025 0.005 0.002

AA 0.024 0.030 0.042 0.006 0.006 0.000

[27]:D10S197

Allele 160 162 164 166 168 170 172 174 176 178 EA 0.000 0.000 0.027 0.004 0.090 0.165 0.152 0.420 0.135 0.005

AA 0.036 0.018 0.048 0.042 0.083 0.220 0.238 0.167 0.113 0.012

Allele 180 182

EA 0.002 0.000

AA 0.018 0.006

[28]:D11S935

Allele 192 196 198 200 202 204 206 208 210 212

EA 0.016 0.002 0.002 0.301 0.028 0.103 0.009 0.069 0.434 0.034

AA 0.000 0.083 0.000 0.190 0.101 0.304 0.202 0.048 0.071 0.000

Allele 214

EA 0.002

AA 0.000

[29]:D9S175

Allele 255 259 261 263 265 267 269 271 273 275 EA 0.002 0.011 0.323 0.152 0.073 0.147 0.034 0.035 0.030 0.050

AA 0.000 0.000 0.077 0.042 0.036 0.196 0.089 0.155 0.131 0.149

Allele 277 279 281 283 285 287 297

EA 0.046 0.057 0.021 0.007 0.011 0.002 0.000

AA 0.054 0.024 0.006 0.000 0.012 0.006 0.024

[30]:D5S410

Allele 325 329 331 333 335 337 339 341 343 345 EA 0.000 0.002 0.103 0.262 0.085 0.381 0.014 0.014 0.103 0.014

AA 0.006 0.012 0.048 0.220 0.464 0.190 0.030 0.012 0.012 0.000

Allele 347 349 351

EA 0.014 0.002 0.005

AA 0.006 0.000 0.000

[31]:D16S3017

Allele 151 155 157 159 161 163 165 167 169 171

EA 0.002 0.000 0.000 0.016 0.209 0.268 0.317 0.096 0.082 0.007

AA 0.000 0.048 0.071 0.036 0.077 0.452 0.143 0.155 0.012 0.006

Allele 173

EA 0.004

AA 0.000

[32]:D10S1786

Allele 183 185 187 189 191 193 195 197 199

EA 0.002 0.002 0.144 0.163 0.012 0.067 0.035 0.523 0.051

AA 0.000 0.149 0.071 0.256 0.042 0.149 0.107 0.149 0.077

[33]:D15S1002

Allele 208 210 212 214 216 218 220 222 224 226 EA 0.027 0.358 0.090 0.009 0.002 0.014 0.018 0.284 0.137 0.041

AA 0.000 0.107 0.048 0.030 0.071 0.292 0.107 0.119 0.113 0.042

Allele 228 230 232 234 236

EA 0.018 0.004 0.000 0.000 0.000

AA 0.042 0.012 0.006 0.006 0.006

[34]:D1S2628

Allele 337 339 341 343 345 347 349 351

EA 0.012 0.300 0.027 0.454 0.073 0.135 0.000 0.000

AA 0.000 0.101 0.149 0.220 0.220 0.256 0.048 0.006

[35]:D12S352

Allele 147 151 155 157 159 161 163 165 167 169

EA 0.000 0.043 0.002 0.078 0.051 0.129 0.489 0.186 0.016 0.005

AA 0.018 0.006 0.006 0.101 0.071 0.268 0.345 0.125 0.054 0.006

[36]:FY^null

Allele 1 2

EA 0.996 0.004

AA 0.173 0.827
